# New insights into the role and signalling processes of gp130

**DOI:** 10.1186/ar3414

**Published:** 2011-09-16

**Authors:** Stefan Rose-John

**Affiliations:** 1 Department of Biochemistry, Christian-Albrechts-Universität, 24098 Kiel, Germany

## 

Cytokine receptors exist in membrane bound and soluble form. While most soluble receptors are antagonists, some soluble receptors are agonists like soluble receptors of the gp130 cytokine family. *In vivo*, the IL-6/soluble IL-6R complex stimulates several types of target cells not stimulated by IL-6 alone, since they do not express the membrane bound IL-6R. This process has been named trans-signaling [1].

We have shown that soluble gp130 is the natural inhibitor of IL-6/soluble IL-6R complex responses. The recombinant soluble gp130 protein is a molecular tool to discriminate between gp130 responses via membrane bound and soluble IL-6R responses. We have constructed a fusion of soluble gp130 and the Fc portion of human IgG1. This sgp130Fc protein proved to be efficient in blocking responses via the IL-6/soluble IL-6R complex without affecting IL-6 responses, which are mediated via the membrane bound IL-6R [1].

The soluble IL-6R is mostly generated by proteolysis of the IL-6R transmembrane protein. Shedding of the IL-6R is mediated by the metalloprotease ADAM17, which is also responsible for the cleavage of TNFα and ligands of the EGF-R. Consequently, activation of ADAM17 has different effects on the activation of the immune response as well as on induction of regenerative responses [2,3].

Interestingly, depending on the animal model used, global blockade of IL-6 signaling by neutralizing monoclonal antibodies and selective blockade of IL-6 trans-signaling can lead to different consequences. We could recently show that inhibition of IL-6 trans-signaling was beneficial in a caecum ligation puncture model whereas global IL-6 blockade showed no benefit in survival of the animals [4]. In contrast, in a sepsis model induced by a bolus injection of LPS, both, global blockade of IL-6 signaling by neutralizing monoclonal antibodies and selective blockade of IL-6 trans-signaling proved effective in preventing the death of the animals [5]. Also various infection models suggest a different outcome of global blockade of IL-6 as compared to selective IL-6 trans-signaling inhibition.

We could show that the extent of IL-6 trans-signaling in chronic inflammatory diseases and cancer is controlled by the soluble IL-6R. Using the sgp130Fc protein or sgp130Fc transgenic mice we demonstrate that in several chronic inflammatory diseases and cancers including inflammatory bowel disease, peritonitis, rheumatoid arthritis, colon cancer, ovarian cancer and pancreatic cancer, that IL-6 trans-signaling via the soluble IL-6R is a crucial step in the development and the progression of the disease. Therefore, sgp130Fc is a novel therapeutic agent for the treatment of chronic inflammatory diseases and cancer [1,6-8].
